# Supplementary material for: An Influx of Non-Native Bird Species into the Natural Environment Owing to the Accidental Release of Pet Birds in Japan
Source: Animals (Basel). 2024 Jan 10;14(2):221. doi: 10.3390/ani14020221 (PMC10812534; doi:10.3390/ani14020221)
Supplement: Supplementary file 1 [file animals-14-00221-s001.zip › animals-2773349-supplementary.pdf]

Supplementary Material

Table S1. The Lost-and-found websites for pets from which the datasets were obtained.

Website	Uniform Resource Locator (URL)
Inko Dayori	https://inkosuki.info/comu/
Toricchi	https://torich.jp/bbs/
Pet no Kimochi	https://petmaigo.net/maigo/bird
Veterinarian Bulletin Board	https://www.vets.ne.jp

Table S2. The lists of invasive birds in Japan and the detected pet bird species. A: Check-list of Japanese birds (7th ed) [23]. B: List of Invasive Species of Japan database [26]. C: Report of the Japan Breeding Bird Atlas [27] (– shows the species is on the list but no observation in the most recent survey). D: Handbook of Introduced Birds in Japan [28]. E: Other articles. * Unconfined keeping of domesticated species or birds in zoos for exhibition. Some may be on the loose without their owners. ** Native subspecies inhabit Japan.

Order	Legal Control	Species	English name	A	B	C	D	E	Escaped
Anseriformes	Yes	*Branta canadensis*	Canada Goose	+	+		+		
Anseriformes		*Anser anser domestica* *	Greylag Goose				+		
Anseriformes		*Anser cygnoides domestica* *	Swan Goose				+		
Anseriformes		*Cygnus atratus*	Black Swan	+	+	+	+		
Anseriformes		*Cygnus olor*	Mute Swan	+	+	+	+		
Anseriformes		*Alopochen aegyptiaca*	Egyptian Goose				+		
Anseriformes		*Cairina moschata domestica* *	Muscovy Duck				+		
Anseriformes		*Aix sponsa*	Wood Duck				+		
Anseriformes		*Anas platyrhynchos domestica* *	Mallard				+		1
Galiformes		*Numida meleagris domestica* *	Helmeted Guineafowl				+		
Galiformes		*Colinus virginianus*	Northern Bobwhite		+	+	+		
Galiformes		Meleagris gallopavo domestica *	Wild Turkey				+		
Galiformes		*Syrmaticus soemmerringii ssp.* **	Copper Pheasant var.	+			+		
Galiformes		*Phasianus colchicus karpowi*	Korean Ring-necked Pheasant	+	+		+		
Galiformes		*Phasianus colchicus ssp.* **	Common Pheasant var.	+			+		
Galiformes		*Pavo cristatus*	Indian Peafowl	+	+	+	+		
Galiformes		*Bambusicola thoracicus*	Chinese Bamboo Partridge	+	+	+	+		
Galiformes		*Bambusicola sonorivox*	Taiwan Bamboo Partridge	+	+	+	+		
Galiformes		*Gallus gallus domestica* *	Red Junglefowl				+		1
Galiformes		*Synoicus chinensis*	King Quail						12
Galiformes		*Coturnix japonica*	Japanese Quail						9
Columbiformes		*Columba livia*	Rock Dove	+	+	+	+		4
Columbiformes		*Streptopelia decaocto*	Eurasian Collared Dove	+		+	+		
Columbiformes		*Streptopelia roseogrisea*	Ringneck Dove						3
Columbiformes		*Geopelia cuneata*	Diamond Dove						2
Charadriiformes		*Himantopus mexicanus*	Black-necked Stilt	+	+		+		
Ciconiiformes		*Mycteria leucocephala* *	Painted Stork				+		
Accipitriformes		*Accipiter gentilis*	Northern Goshawk						3
Accipitriformes		*Parabuteo unicinctus*	Harris's Hawk						6
Strigiformes		*Tyto spp.*	Barn Owl						8
Strigiformes		*Athene noctua*	Little Owl						3
Strigiformes		*Bubo bengalensis*	Indian Eagle-Owl						1
Strigiformes		*Bubo africanus*	Spotted Eagle-Owl						2
Strigiformes		*Ketupa ketupu*	Buffy Fish Owl						1
Strigiformes		*Strix rufipes*	Rufous-legged Owl						1
Strigiformes		*Strix aluco*	Tawny Owl						2
Falconiformes		*Falco tinnunculus*	Common Kestrel						2
Falconiformes		*Falco sparverius*	American Kestrel						1
Falconiformes		*Falco cherrug*	Saker Falcon						1
Falconiformes		*Falco peregrinus*	peregrine Falcon						2
Falconiformes		*Nymphicus hollandicus*	Cockatiel						2750
Psittaciformes		*Eolophus roseicapilla*	Galah						9
Psittaciformes		*Cacatua galerita*	Sulphur-crested Cockatoo						3
Psittaciformes		*Cacatua alba*	White Cockatoo						2
Psittaciformes		*Psittacus erithacus*	Grey Parrot						46

Psittaciformes		*Poicephalus gulielmi*	Red-fronted Parrot						4
Psittaciformes		*Poicephalus meyeri*	Meyer's Parrot						3
Psittaciformes		*Poicephalus senegalus*	Senegal Parrot						2
Psittaciformes		*Poicephalus sp.*	Poicephalus Parrot						1
Psittaciformes		*Bolborhynchus lineola*	Barred Parakeet						100
Psittaciformes		*Myiopsitta monachus*	Monk Parakeet	+	+	–	+		71
Psittaciformes		*Brotogeris versicolurus*	White-winged Parakeet						1
Psittaciformes		*Pionus menstruus*	Blue-headed Parrot						3
Psittaciformes		*Amazona aestiva*	Turquoise-fronted Amazon					[30]	1
Psittaciformes		*Amazona sp.*	Amazon sp						1
Psittaciformes		*Forpus coelestis*	Pacific Parrotlet						136
Psittaciformes		*Pionites melanocephalus*	Black-headed Parrot						7
Psittaciformes		*Pionites leucogaster*	White-bellied Parrot						7
Psittaciformes		*Deroptyus accipitrinus*	Red-fan Parrot						1
Psittaciformes		*Pyrrhura spp.*	Pyrrhura spp.						228
Psittaciformes		*Aratinga weddellii*	Dusky-headed Parakeet						4
Psittaciformes		*Aratinga solstitialis*	Sun Parakeet						40
Psittaciformes		*Aratinga jandaya*	Jandaya Parakeet						7
Psittaciformes		*Ara ararauna*	Blue-and-yellow Macaw						13
Psittaciformes		*Ara severus*	Chestnut-fronted Macaw						1
Psittaciformes		*Ara macao*	Scarlet Macaw						2
Psittaciformes		*Diopsittaca nobilis*	Red-shouldered Macaw						12
Psittaciformes		*Ara sp.*	Macaw hybrid						1
Psittaciformes		*Polytelis anthopeplus*	Regent Parrot						1
Psittaciformes		*Alisterus scapularis*	Australian King Parrot						1
Psittaciformes		*Eclectus roratus*	Eclectus Parrot						2
Psittaciformes		*Psittacula cyanocephala*	Plum-headed Parakeet						1
Psittaciformes		*Psittacula alexandri*	Red-breasted Parakeet	+	+	+	+		3
Psittaciformes		*Psittacula derbiana*	Lord Derby's Parakeet						1
Psittaciformes		*Psittacula eupatria*	Alexandrine Parakeet	+	+		+		
Psittaciformes		*Psittacula krameri*	Rose-ringed Parakeet	+	+	+	+		14
Psittaciformes		*Psephotus haematonotus*	Red-rumped Parrot						7
Psittaciformes		*Platycercus elegans*	Crimson Rosella						1
Psittaciformes		*Platycercus eximius*	Eastern Rosella						2
Psittaciformes		*Neopsephotus bourkii*	Bourke's Parrot						38
Psittaciformes		*Neophema pulchella*	Turquoise Parrot						2
Psittaciformes		*Saudareos ornatus*	Ornate Lorikeet						1
Psittaciformes		*Trichoglossus spp.*	Lorikeet						5
Psittaciformes		*Melopsittacus undulatus*	Budgerigar	+	+	–	+		6469
Psittaciformes		*Agapornis roseicollis*	Rosy-faced Lovebird						801
Psittaciformes		*Agapornis spp.*	Lilian's Lovebird						227
Passeriformes		*Urocissa caerulea*	Taiwan Blue Magpie	+	+	–	+		
Passeriformes		*Urocissa erythroryncha*	Red-billed Blue Magpie			+		[29]	
Passeriformes		*Pica pica*	Eurasian Magpie	+	+		+		
Passeriformes		*Pycnonotus sinensis ssp.* **	Light-vented Bulbul		+		+		
Passeriformes		*Pycnonotus jocosus*	Red-whiskered Bulbul		+		+		
Passeriformes		*Zosterops japonicus ssp.* **	Warbling White-eye	+			+		
Passeriformes	Yes	*Leiothrix lutea*	Red-billed Leiothrix	+	+	+	+		
Passeriformes	Yes	*Garrulax canorus*	Chinese Hwamei	+	+	+	+		
Passeriformes	Yes	*Ianthocincla cineracea*	Moustached Laughingthrush	+	+	+	+		
Passeriformes	Yes	*Pterorhinus sannio*	White-browed Laughingthrush	+	+	+	+		
Passeriformes	Yes	*Pterorhinus perspicillatus*	Masked Laughingthrush	+	+	+	+		
Passeriformes		*Gracula religiosa*	Common Hill Myna		+				3
Passeriformes		*Acridotheres cristatellus*	Crested Myna	+	+	+	+		
Passeriformes		*Acridotheres fuscus*	Jungle Myna	+	+		+		
Passeriformes		*Acridotheres ginginianus*	Bank Myna	+			+		
Passeriformes		*Acridotheres tristis*	Common Myna	+		+	+		
Passeriformes		*Gracupica contra*	Indian Pied Myna	+	+		+		
Passeriformes		*Ploceus intermedius*	Lesser Masked Weaver	+	+		+		
Passeriformes		*Ploceus manyar*	Streaked Weaver		+		+		
Passeriformes		*Euplectes afer*	Yellow-crowned Bishop		+		+		
Passeriformes		*Euplectes orix*	Southern Red Bishop	+	+	–	+		
Passeriformes		*Taeniopygia guttata*	Zebra Finch						60
Passeriformes		*Padda oryzivora*	Java Sparrow	+	+	–	+		946
Passeriformes		*Lonchura punctulata*	Scaly-breasted Munia	+	+	+	+		
Passeriformes		*Lonchura striata*	White-rumped Munia	+	+	–	+		
Passeriformes		*Lonchura striata domestica*	Bengalese finch						10
Passeriformes		*Lonchura malacca*	Tricolored Munia	+	+	+	+		

Passeriformes	*Lonchura atricapilla*	Chestnut Munia	+	+	+	+	
Passeriformes	*Lonchura maja*	White-headed Munia	+	+	–	+	
Passeriformes	*Chloebia gouldiae*	Gouldian Finch					3
Passeriformes	*Estrilda melpoda*	Orange-cheeked Waxbill	+	+		+	
Passeriformes	*Estrilda troglodytes*	Black-rumped Waxbill	+	+		+	
Passeriformes	*Amandava amandava*	Red Avadavat	+	+	–	+	
Passeriformes	*Vidua macroura*	Pin-tailed Whydah		+		+	
Passeriformes	*Vidua paradisaea*	Long-tailed Paradise Whydah	+	+			
Passeriformes	*Serinus canaria domestica*	Domestic Canary					6
Passeriformes	*Paroaria coronata*	Red-crested Cardinal	+	+	–	+	
